# Insights into the evolution of Darwin’s finches from comparative analysis of the *Geospiza magnirostris* genome sequence

**DOI:** 10.1186/1471-2164-14-95

**Published:** 2013-02-12

**Authors:** Chris M Rands, Aaron Darling, Matthew Fujita, Lesheng Kong, Matthew T Webster, Céline Clabaut, Richard D Emes, Andreas Heger, Stephen Meader, Michael Brent Hawkins, Michael B Eisen, Clotilde Teiling, Jason Affourtit, Benjamin Boese, Peter R Grant, Barbara Rosemary Grant, Jonathan A Eisen, Arhat Abzhanov, Chris P Ponting

**Affiliations:** 1 Department of Physiology, Anatomy, and Genetics, MRC Functional Genomics Unit, University of Oxford, Oxford, OX1 3PT, UK; 2 UC Davis Genome Center, University of California Davis, Davis, CA, USA; 3 Harvard University, Organismic and Evolutionary Biology, Cambridge, MA, 02138-2020, USA; 4 Science for Life Laboratory, Department of Medical Biochemistry and Microbiology, Uppsala University, Uppsala, 751 23, Sweden; 5 School of Veterinary Medicine and Science, University of Nottingham, Leicestershire, LE12 5RD, UK; 6 Advanced Data Analysis Centre, University of Nottingham, Nottingham, UK; 7 Department of Molecular and Cell Biology, University of California Berkeley, Berkeley, CA, USA; 8 Howard Hughes Medical Institute, University of California Berkeley, Berkeley, CA, USA; 9 454 Life Sciences, a Roche Company, Branford, CT, USA; 10 Life Technologies, South San Francisco, CA, USA; 11 Princeton University, Ecology and Evolutionary Biology, Princeton, NJ, 08544-2016, USA; 12 Department of Evolution and Ecology, University of California Davis, Davis, CA, USA; 13 Department of Medical Microbiology and Immunology, University of California Davis, Davis, CA, USA

**Keywords:** Genomics, Evolution, Darwin’s finches, Large ground finch, *Geospiza magnirostris*

## Abstract

**Background:**

A classical example of repeated speciation coupled with ecological diversification is the evolution of 14 closely related species of Darwin’s (Galápagos) finches (Thraupidae, Passeriformes). Their adaptive radiation in the Galápagos archipelago took place in the last 2–3 million years and some of the molecular mechanisms that led to their diversification are now being elucidated. Here we report evolutionary analyses of genome of the large ground finch, *Geospiza magnirostris*.

**Results:**

13,291 protein-coding genes were predicted from a 991.0 Mb *G. magnirostris* genome assembly. We then defined gene orthology relationships and constructed whole genome alignments between the *G. magnirostris* and other vertebrate genomes. We estimate that 15% of genomic sequence is functionally constrained between *G. magnirostris* and zebra finch. Genic evolutionary rate comparisons indicate that similar selective pressures acted along the *G. magnirostris* and zebra finch lineages suggesting that historical effective population size values have been similar in both lineages. 21 otherwise highly conserved genes were identified that each show evidence for positive selection on amino acid changes in the Darwin's finch lineage. Two of these genes (*Igf2r* and *Pou1f1*) have been implicated in beak morphology changes in Darwin’s finches. Five of 47 genes showing evidence of positive selection in early passerine evolution have cilia related functions, and may be examples of adaptively evolving reproductive proteins.

**Conclusions:**

These results provide insights into past evolutionary processes that have shaped *G. magnirostris* genes and its genome, and provide the necessary foundation upon which to build population genomics resources that will shed light on more contemporaneous adaptive and non-adaptive processes that have contributed to the evolution of the Darwin’s finches.

## Background

"“The most curious fact is the perfect gradation in the size of the beaks in the different species of Geospiza, from one as large as that of a hawfinch to that of a chaffinch, and… even to that of a warbler… Seeing this gradation and diversity of structure in one small, intimately related group of birds, one might really fancy that from an original paucity of birds in this archipelago, one species had been taken and modified for different ends.”"

Charles R. Darwin, The Voyage of the Beagle [1]

Since their collection by Charles Darwin and fellow members of the HMS Beagle expedition from the Galápagos Islands in 1835 and their introduction to science, these birds have been subjected to intense research. Many biology textbooks use Darwin’s finches (formerly known as Galápagos finches) to illustrate a variety of topics in evolutionary theory, including speciation, natural selection, and niche partitioning [2-4]. Darwin’s finches continue to be a very valuable source of biological discovery. Several unique characteristics of this clade have allowed multiple important recent breakthroughs in our understanding of changes in island biodiversity, mechanisms of repeated speciation coupled with ecological diversification, evolution of cognitive behaviours, principles of beak/jaw biomechanics as well as the underlying developmental genetic mechanisms in generating morphological diversity [5,6].

**Figure 1:**
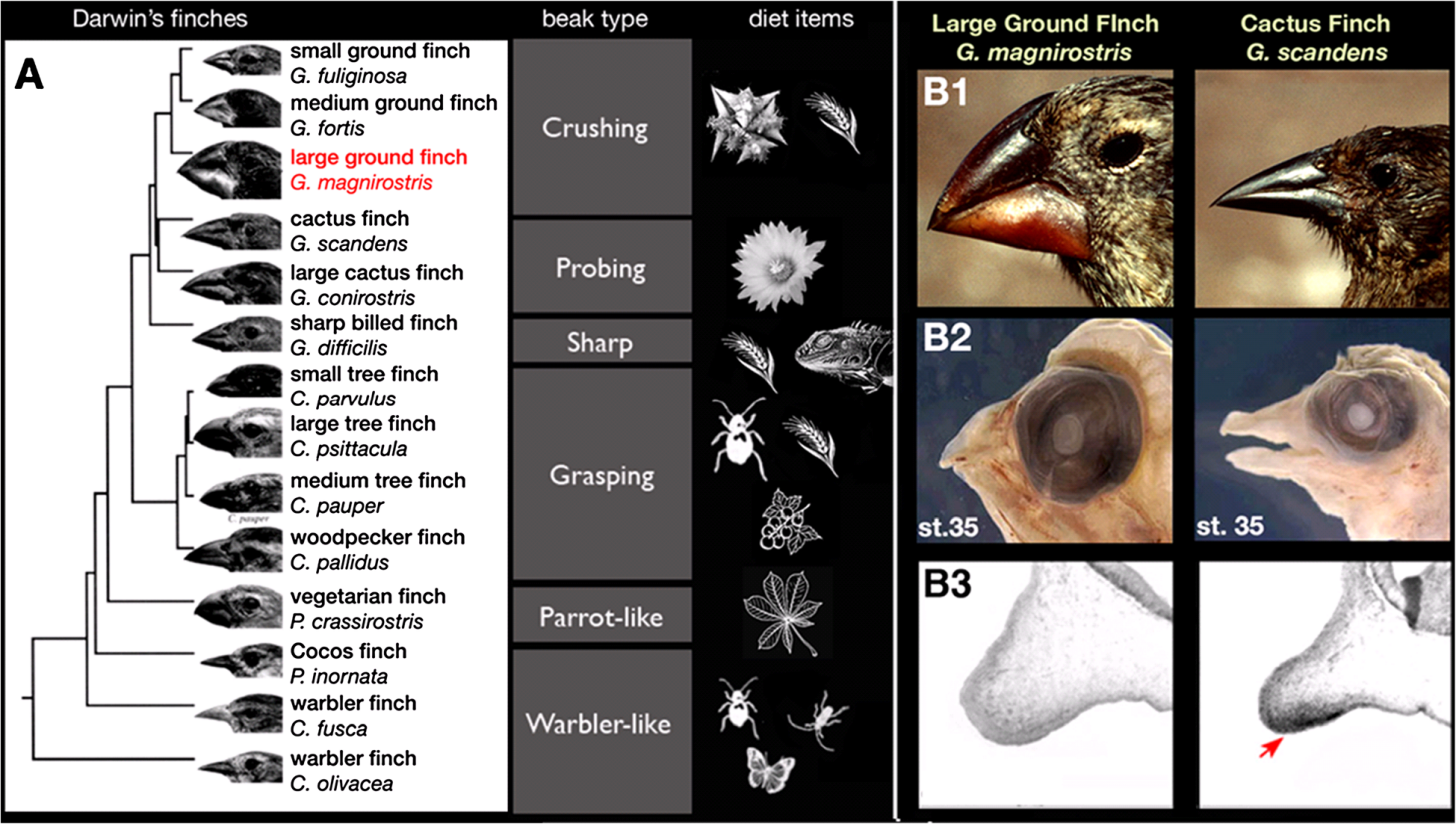
**Evolutionary mechanisms for beak shape diversity in Darwin’s finches (Thraupidae, Passeriformes).** (**A**) Molecular phylogeny of 14 species of Darwin’s finches shows a range of beak shapes in this group of birds. These species have beaks of different shapes that allow them to feed on many different diets: insects, seeds, berries, and young leaves. Species are numbered as follows: small ground finch *Geospiza fuliginosa*; medium ground finch *G. fortis*; large ground finch *G. magnirostris*; cactus finch *G. scandens*; large cactus finch *G. conirostris*; sharp-billed finch *G. difficilis*; small tree finch *C. parvulus*; large tree finch *Camarhynchus psittacula*; medium tree finch *C. pauper*; woodpecker finch *C. pallidus*; vegetarian finch *Platyspiza crassirostris*; Cocos finch *Pinaroloxias inornata*; warbler finch *Certhidea fusca*; warbler finch *C. olivacea* (phylogeny from [5]). (**B1**) Large ground finch (left) has a very deep and broad bill adapted to crack hard and large seeds, while the cactus finch (right) has an elongated and pointy beak for probing cactus flowers and fruits. (**B2**) *Geospiza* finch bills develop their distinct shapes during embryogenesis and are apparent upon hatching (mid-development stage 35 embryos are shown from Abzhanov *et al.*[12]). (**B3**) The cactus finch-specific expression of CaM was validated by in situ hybridization after it was identified as a candidate by a microarray screen [14].

Recent molecular phylogenetic reconstructions suggest that the adaptive radiation of Darwin’s finches in the Galápagos archipelago took place in the last 2–3 million years (my), following their evolution from a finch-like tanager ancestral species that probably arrived on the islands from Central or South America (Figure 1; [7-9]). Nuclear microsatellite and mitochondrial DNA have undergone limited diversification, partly because the Galápagos history of the finches has been relatively short, and partly because of introgressive hybridization [10,11]. Morphological evolution in this group of birds is a fast and ongoing process that has been documented over the years in multiple publications on their population-level ecology, morphology and behaviour [5].

Beak size and shape, as well as body size, are the principal phenotypic traits that have diversified in Darwin’s finches [5]. The most studied group within the Darwin’s finches is the monophyletic genus *Geospiza*, which includes three distinct bill shapes: the basal sharp-billed finch *G. difficilis* has a small and symmetrical beak used to feed on a mixed diet of insects and seeds; cactus finches *G. scandens* and *G. conirostris* feature an elongated and pointed bill suitable for probing cactus flowers and fruit; and ground finches possess deep and broad bills adapted for cracking seeds [5]. Among the ground finches, which include small, medium and large species, the large ground finch *G. magnirostris* has the most modified beak that it uses to crack (and then consume) large and hard seeds (Figure 1). Importantly, beak shapes develop during early embryogenesis and finch hatchlings show species-specific features. Recent molecular analysis has shown that the ground finch bill morphology correlates with a developmentally earlier and broader gene expression of *Bone morphogenetic protein 4* (*Bmp4*), especially in the large ground finch. Functional experiments mimicking such changes in *Bmp4* expression using laboratory chicken embryos are consistent with its role in this *Geospiza* beak trait [12]. Similar experiments elucidated the roles of three further developmental factors, *Transforming Growth Factor beta Receptor Type II* (*TGFβRII*), *beta-Catenin* (*βCat*) and *Dickkopf-3* (*Dkk3*), at later stages of beak development that help in forming the bill shapes that are unique to ground finches [13]. Other analyses revealed an important role of change in *Calmodulin* (*CaM*) expression pattern for the development of elongated bills of cactus finches [14].

In 2008 we initiated a project to sequence the genomes of some of the Darwin’s finches (Additional file 1). In particular, we were motivated to perform a whole genome analysis of the large ground finch *G. magnirostris* because of the evolutionary importance of the entire clade of Darwin’s finches to the fields of ecology and evolutionary biology, the potential of genomic analysis for uncovering the genetic basis of key phenotypic traits and the scarcity of genomic studies of birds (especially when compared to mammals). The species was chosen because it arose relatively recently and it has one of the most adapted and distinctive bill shapes. The embryonic individual chosen for genome sequencing was sampled from a population from the small and well isolated island of Genovesa which exhibit the largest bills of all existing Darwin’s finches, with an estimated effective population size of 75–150 individuals [5].

The field of evolutionary and comparative genomics will benefit more broadly from analyzing an additional species of passerine. *G. magnirostris* diverged from the first sequenced passerine, the zebra finch (*Taeniopygia guttata*) [15], approximately 25 my ago [16], which is comparable to the divergence time separating mouse and rat [17]. The *G. magnirostris* genome assembly has not been assembled into chromosomes or long contigs so we cannot investigate whether this interval of time has seen radical changes in its karyotype; however, such changes are unlikely since avian karyotypes typically are stable [18,19]. Nevertheless, we can investigate a variety of other evolutionary processes, such as whether episodes of positive selection have occurred along the *G. magnirostris* terminal lineage and whether there has been rapid gains and losses (evolutionary ‘turnover’ [20]) of functional sequence across the avian clade.

The genome assembly and analysis presented here should permit population genetics approaches to be applied to Darwin’s finch species and subpopulations in order to identify the genetic basis of their recent adaptations.

## Results and discussion

### A *G. magnirostris* genome assembly

A DNA sample was taken from a *G. magnirostris* individual embryo collected during a field trip to the island of Genovesa (Galápagos) in 2009. Sequencing was performed using the Roche 454 technology with both long read and mate-pairs libraries, and then assembled using Roche’s algorithm Newbler, as described in the Materials and Methods. The resulting assembly contains 991.0 Mbp across 12,958 scaffolds with a scaffold N50 of 382kbp and a median read coverage of 6.5-fold.

Completeness of the *G. magnirostris* genome assembly was estimated using two approaches. First, we determined the amount of euchromatic sequence that aligns between zebra finch and chicken, but that does not align to *G. magnirostris*. Since chicken is an outgroup to both zebra finch and *G. magnirostris*, we can assume that most sequence present in both the zebra finch and chicken genome assemblies will also be present in the *G. magnirostris* assembly, with rare exceptions where lineage-specific deletions have occurred along the Darwin's finch lineage. Thus, the 122 Mb of chicken sequence aligned to zebra finch that is absent from the *G. magnirostris* assembly provides an estimate of the *G. magnirostris* euchromatic genome assembly’s incompleteness. Second, the assembly consists of approximately 7.529 Gb of sequence data, and the depth of coverage for reads on assembled contigs peaks at 6.0. Consequently, under a simplifying assumption that all regions of the genome are equally represented in libraries and among successful sequencing runs, an estimate of the true genome size is 7.529/6.0 or 1.25 Gb. In summary, the *G. magnirostris* genome assembly is estimated to cover approximately 89% of the euchromatic genome or approximately 76% of the complete genome. The estimated 1.250 Gb size of the *G. magnirostris* genome is similar to the mean avian genome size (1.38Gb, [21]. Animal Genome Size Database. http://www.genomesize.com).

We expect this *G. magnirostris* genome assembly to be most incomplete within highly repetitive sequence. Use of either a library of transposable element sequences constructed from the *G. magnirostris* genome (using RepeatScout [22]) or a zebra finch repeat library resulted in the identification of 3.3% or 4.1% of the assembly as being repetitive, respectively. This proportion is over two-fold lower than observed for zebra finch or chicken genomes [15,23], and it is clear that there is a deficit of closely-related transposable elements present in the *G. magnirostris* assembly (Additional file 2). Highly repetitive sequence in the *G. magnirostris* genome is thus likely to be disproportionately missing from the assembly.

Assembly sequence quality was assessed first by examining whether GT-AG dinucleotide splice sites in 6,188 chicken genes, each with a single orthologue in zebra finch and *G. magnirostris*, exhibited apparently substituted nucleotides in aligned *G. magnirostris* sequence. 515 of 168,849 (0.31%) of these nucleotides showed sequence changes, providing an estimate of the assembly’s nucleotide substitution errors. Although this is higher than error rates inferred in other sequenced avian genomes, such as the 0.05% rate estimated for zebra finch [15], it is likely to overestimate the true error rate, because some substitutions will reflect mis-alignments or genuine point mutations. In a second approach, we counted the number of insertions or deletions (‘indels’) that are present in the three-way alignment of zebrafinch with *G. magnirostris* and a *G. fortis* sequence that was recently released (GenBank entry: AKZB00000000.1 [24]). If one conservatively assumes that there have been no *G. magnirostris* lineage-specific indels then the upper-bound estimate for the indel error is 1.98 indels per Kb of aligned sequence. These errors will have led to a lowering of the number of protein-coding gene models that we predict for *G. magnirostris.*

These approaches took advantage of whole genome alignments constructed for *G. magnirostris* and chicken, zebra finch and turkey. 57% of the *G. magnirostris* assembly aligned to chicken and 58% to turkey (Table 1), which is similar to the 58% and 56% of the zebra finch assembly that aligned to chicken and turkey, respectively [25]. A large proportion (83%) of the Darwin’s finch genome could be aligned to zebra finch (Table 1), consistent with their more recent ancestry than with chicken or turkey, which are both galliforms.

**Table 1:** **Amount of sequence aligning between*G. magnirostris*and genome assemblies from other avian species**

**Species Pair**	**Genome Size (Mb)**		**Aligning sequence (Mb)**	**Percentage of the*G. magnirostris*genome aligning (%)**
	**First species**	**Second species**		
*G. gallus* – *G. magnirostris*	1037	991	569	57
*M. gallopavo* – *G. magnirostris*	1046	991	578	58
*T. guttata* – *G. magnirostris*	1058	991	823	83

The *G. magnirostris* genome assembly has a G+C proportion of 40.08%, which is similar to all other evaluated amniote genomes. Medium-sized scaffolds (sizes between 2398 bp and 46677 bp) were more G+C-rich (44.6%) than small or large scaffolds (41.2% and 39.8%, respectively). Visual inspection of the *G. magnirostris* genome reveals that it exhibits substantial spatial heterogeneity in its base composition; similarly to all other amniotic genomes, but unlike that of the *Anolis* lizard [26], genic G+C content of genomic regions has remained relatively constant (Additional file 3).

### Neutral indel model analysis

The Neutral Indel Model (NIM) of Lunter *et al.*[27] provides an estimate of the amount of sequence that has been functionally constrained in one or both members of a species pair since their last common ancestor. The method takes advantage of an expectation that autosomal indels in a genome-wide pairwise sequence alignment occur randomly once account has been taken of fluctuations in G+C content. Where their density is relatively low it is assumed that there is a greater likelihood that additional insertion or deletion variants have been preferentially purged in functional sequence. The NIM first constructs histograms of the lengths of inter-gap segments (IGSs; defined as ungapped segments of aligned sequence between a species pair) from whole genome pairwise alignments, and then measures the departure of the observed IGS frequency distribution from the random distribution expected under neutral evolution. The excess of long IGSs compared to the neutral expectation allows the quantity of constrained, indel-purified, sequence shared between the two species to be inferred.

**Figure 2:**
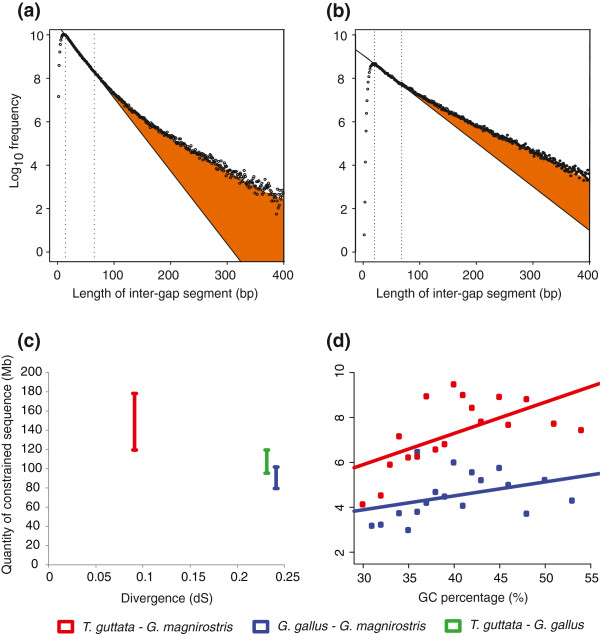
**Constrained sequence analyses.** Frequency histograms of inter-gap segment lengths are compared against the neutral expectation (solid line) (**a**,**b**). The shaded orange area represents the total amount of indel-purified sequence shared by the species pair. Histograms are derived from (**a**) chicken - *G. magnirostris* and (**b**) chicken - zebra finch whole genome alignments. Results are shown for a representative G+C-fraction from the 11^th^ of 20 equal size G+C-bins, with the corresponding histograms from all G+C-fractions presented in Additional file 9, Additional file 10 and Additional file 11. Predicted amounts of constrained sequence estimated between three avian species pairs plotted against (**c**) the synonymous substitution divergence (*d*_*S*_) and (**d**) GC content of equally populated GC bins, with data inferred from the *T. guttata* – *G. magnirostris* and *G. gallus – G. magnirostris* alignments, respectively. The larger amount of constrained sequence inferred for the *G. magnirostris* and *T. guttata* comparison compared to the two chicken – finch comparisons implies that there is functional sequence that is passerine-specific and thus not present in chicken.

The NIM method estimates there to be 80–120 Mb of constrained sequence between chicken and *G. magnirostris*, similar to the amount of constrained sequence (96–120 Mb) estimated between the comparably divergent chicken and zebra finch species (Figure 2a, b). However, these estimates are substantially smaller than the amount of constrained sequence estimated between zebra finch and *G. magnirostris* (120–179 Mb) (Figure 2c). Since zebra finch and *G. magnirostris* are more closely related than either is to chicken, these results are consistent with the loss of shared functional sequence over avian evolution, and the gain of lineage-specific functional sequence, as has been inferred previously in mammals [20,28]. It is notable also that the lower bound estimate of sequence constraint is far in excess of the quantity of protein-coding sequence (approximately 29 Mb) in avian genomes, implying that the majority of functional sequence in avian genomes is noncoding, probably regulatory, sequence. As has been observed for eutherian mammals [29], genomic regions with elevated G+C content tend to contain a higher density of constrained sequence (Figure 2d).

### *G. magnirostris* predicted genes and orthologues

We predicted 13,291 protein-coding genes in the *G. magnirostris* genome assembly. To do so we aligned protein-coding sequences from three amniote species, human, chicken, and zebra finch, to the *G. magnirostris* genome assembly, and reconciled overlapping transcript predictions using the Gpipe pipeline [30]. To analyse the evolution of *G. magnirostris* protein-coding genes, the orthologues and paralogues among *G. magnirostris* and seven other Euteleostomi (human, mouse, chicken, turkey, zebra finch, *Anolis* lizard and tetraodon) were assigned using the OPTIC pipeline [30,31]. We then produced a high quality set of 1,452 simple orthologue sets (genes that have been spared from duplication or deletion in the bird, reptile and mammalian lineages since their last common ancestor) among the seven amniote species. These 1,452 gene sets represent a stringent set of evolutionarily conserved “core” protein-coding genes in vertebrates.

Examining the completeness of these gene sets, we noted that there were 10,222 simple 1:1 orthologue sets between human and zebra finch, while there were only 7,416 simple 1:1 orthologue sets between human and *G. magnirostris*. The smaller gene orthologue set between human and *G. magnirostris* could imply that 27% of genes are missing from the gene set, and thus the gene set could be 73% complete. A similar proportion (71%) of 1,109 metazoan single copy orthologues curated by Creevey *et al.*[32] have orthologues among our predicted *G. magnirostris* genes. Our approaches ensure that each gene in these orthologue sets has at least one transcript that covers at least 80% of the human, chicken or zebrafinch template transcript. We note that these gene set completeness estimates are lower-bound estimates for assembly completeness since this orthology analysis will exclude some partially, imperfectly or fragmentary predicted *G. magnirostris* gene models.

### Evolutionary rate analysis

**Figure 3:**
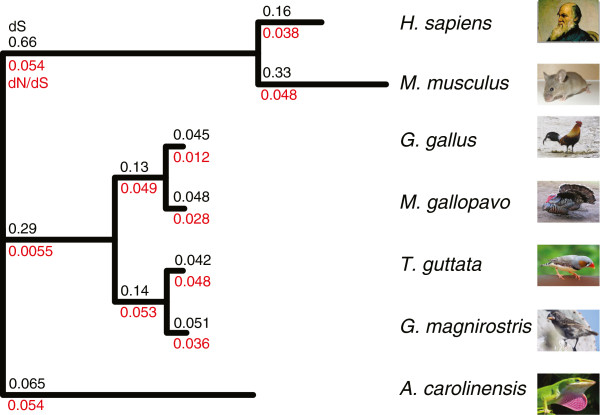
**Phylogeny of seven amniotic species.** Branch lengths are proportional to *d*_*S*_; the degree of constraint (*d*_*N*_*/d*_*S*_) for each terminal lineage is also indicated (values shown in red). Evolutionary rates (*d*_*S*_ and *d*_*N*_*/d*_*S*_) are median values deriving from 1,452 alignments of simple one-to-one orthologues present in each species.

Evolutionary rates (*d*_*S*_, *d*_*N*_, and *d*_*N*_*/d*_*S*_ values) were inferred for the filtered alignments for the 1,452 sets of orthologues for seven amniote species (Figure 3). The median *d*_*S*_ value for the *G. magnirostris* lineage (0.051) is over 15-fold larger than our predicted nucleotide error rate (0.31%; see above), which indicates that sequencing errors will have little effect on most of our comparative genomic analyses. The estimated median *d*_*S*_ value between zebra finch and *G. magnirostris* (*d*_*S*_ = 0.093) is similar to that for chicken and turkey. Divergence of chicken and turkey lineages occurred approximately two-fold earlier (estimated at 44–59 my ago from mitochondrial and *cyt b* DNA sequences using a Bayesian framework informed by fossil data [33]) than the presumed zebra finch and *G. magnirostris* lineages split (approximately 25 my ago). This implies that neutral evolution was approximately two-times faster in the zebra finch and *G. magnirostris* lineages than in the chicken and turkey lineages, which is consistent with previous findings [34]. A similarly elevated neutral evolutionary rate observed for the rodent lineage has been ascribed to their shorter generation times and their greater rate of DNA replication errors during germ cell division [35]. The generation time of chicken (approximately 2 years [36]) is shorter than that of extant *Geospiza* species (approximately 4.5-5.7 years based on estimates from *G. scandens* and *G. fortis*[37,38]). Nevertheless, the relatively rapid rate of neutral evolution for the zebra finch or *G. magnirostris* lineages would be consistent with historic generation times, over the last 25 million years, for their ancestral species being much shorter than for extant ones.

The lineage-specific median *d*_*N*_*/d*_*S*_ value is slightly smaller for *Geospiza* than it is for zebra finch (Figure 3). Smaller *d*_*N*_*/d*_*S*_ values are expected for lineages with larger effective population sizes *N*_*e*_[39], which implies that since the last common ancestor of zebra finch and *G. magnirostris* historic *N*_*e*_ values have been high, far higher than the very low *N*_*e*_ values of 38–60 of extant *Geospiza* species [40] and closer to the current effective population size of zebra finch (25,000 – 7,000,000) [41].

**Figure 4:**
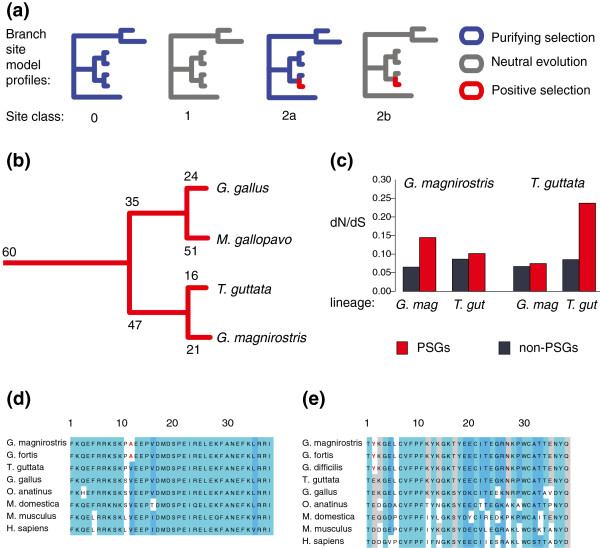
**Evolutionary rate analyses.** (**a**) The Branch-site test models of Zhang *et al.*[42]. The schematic represents the alternative model that allows for positive selection. Under the null model, sites fall into site classes 0 or 1 only. The two models are compared using a likelihood ratio test. (**b**) The number of positively selected genes identified on *G. magnirostris*, *T. guttata*, passerine, *G. gallus*, *M. gallopavo*, galliform, and avian branches. (**c**) Average levels of dN/dS for the *G. magnirostris* or *T. guttata* lineages for *G. magnirostris* and *T. guttata* positively-selected genes (PSGs) and for non-PSGs inferred by parsimony. Alignment showing the candidate *Geospiza* positively selected codon sites (highlighted in red) in (**d**) POU1F1 and (**e**) IGF2R. Alignment visualised with the belvu software [90].

For each of the 1,452 sets of orthologs we next inferred amino acid sites that evolved under positive selection along the *G. magnirostris* lineage, and each of the other three avian lineages. For this we used a branch-sites method [42] and a Bayes Empirical Bayes approach [43] to predict sites that evolved under positive selection (those with a posterior probability > 95% of falling in a site class where *d*_*N*_*/d*_*S*_ = ω >1 along a defined branch; Figure 4a). This procedure resulted in predicting 21, 16, 24 and 51 positively-selected genes (PSGs) in *G. magnirostris,* zebra finch, chicken and turkey lineages, respectively (Figure 4b). This is far fewer than reported previously in avian genomes [44], which likely reflects the lower number of genes that we analysed, the fact these genes are from a more widely conserved orthologue set, and the stringent filters on aligned sites that we needed to employ to discard potentially misaligned or poor quality sequence. Three of the *G. magnirostris* PSGs (Ubiquitin carboxyl-terminal hydrolase; Ubiquitin carboxyl-terminal hydrolase 47; and *IGF2R*) may have been subject to GC-biased gene conversion [45] as indicated from their relatively high numbers of AT→GC substitutions (Additional file 4).

Genes that are predicted to have been under positive selection in the *G. magnirostris* lineage have elevated values of *d*_*N*_/*d*_*S*_ in that lineage, but not the *T. guttata* lineage, and vice versa (Figure 4c). Of the 21 *G. magnirostris* PSGs (Table 2), three were identified as PSGs in other avian lineages: xanthine dehydrogenase (*XDH*), perhaps as a result of its role in the innate immune system [46], mitochondrial ATP binding cassette (ABC) transporter, *ABCB10*, which is essential for erythropoiesis [47] and nebulin (*NEB*), which encodes a large muscle protein [48].

**Table 2:** Positively selected genes along the Darwin’s finch lineage

**Short gene name**	**Ensembl gene ID of chicken 1:1 ortholog**	**P-value that gene is under positive selection**	**Number of codon sites inferred to be under positive selection with p<0.1**
*FKBP6*	ENSGALG00000000837	0.045	0
*MFF*	ENSGALG00000003079	0.030	3
*ASB6*	ENSGALG00000004378	0.048	3
*SART3*	ENSGALG00000004887	0.026	1
*UBP47*	ENSGALG00000005569	**0.0080**	0
*TRAF7*	ENSGALG00000005767	0.021	1
*XDH*	ENSGALG00000008701	**0.0036**	1
*E1BY77*	ENSGALG00000008909	**0.0025**	2
*F1N8A7*	ENSGALG00000010043	**0.0024**	1
*P2RY1*	ENSGALG00000010357	0.022	0
*F1NDU4*	ENSGALG00000011096	0.026	0
*PRKAG3*	ENSGALG00000011360	0.034	1
*ANO10*	ENSGALG00000011513	**0.0038**	1
*IGF2R*	ENSGALG00000011621	0.031	0
*F1NIP9*	ENSGALG00000012138	0.018	1
*LRR1*	ENSGALG00000012230	0.044	1
*C7orf25*	ENSGALG00000012333	0.020	0
*Q9DEH4*	ENSGALG00000012495	**0.0069**	0
*ARSK*	ENSGALG00000014672	**0.0061**	1
*F1NR67 (POU1F1)*	ENSGALG00000015495	0.016	1
*E1BV11*	ENSGALG00000016811	**0.0038**	1

P-values of less than 0.01 are highlighted in bold.

Two *G. magnirostris* PSGs are of particular note: *POU1F1* (POU domain, class 1, transcription factor 1; also known as Pit1, growth hormone factor 1) and *IGF2R* (insulin-like growth factor 2 receptor). These genes’ putatively adaptive amino acid substitutions were confirmed using sequence data from *G. fortis* (medium ground finch) [24] and from *G. difficilis* (sharp-beaked ground finch) (Figure 4d, e). Disruption of either gene in the mouse is known to result in craniofacial abnormalities [49,50] and *POU1F1*, despite its description as a pituitary-specific transcription factor in mammals [51], is differentially expressed in the developing beaks of ducks, quails and chickens [52]. There is a functional link between these two genes since *POU1F1* regulates prolactin and growth hormone genes in mammals and birds [53], and decreased growth hormone results in a decrease in activity of the insulin/IGF-1 signalling pathway [54]. In mouse bone, growth hormone is known to regulate many genes of the *insulin*/*IGF-1* or *Wnt* signaling pathways, as well as *Bmp4*[55] whose gene expression change is linked to bill morphology in *G. magnirostris*[12]. Moreover, a key member of the *IGF* pathway (IGF binding protein, a molecule that controls ligand-receptor interaction) was identified in Darwin’s finches as one of the top differentially expressed candidate genes in a microarray screen in species with divergent beak shapes [14]. Positive selection acting on *POU1F1* and *IGF2R* may thus have contributed to the evolution of beak morphology in the *G. magnirostris* lineage. Experiments that misexpress *POU1F1* or *IGF2R* variants during avian craniofacial development will be required to further investigate this hypothesis.

We also predicted 47 genes to have been under positive selection on the passerine branch prior to the split of the zebra finch and *G. magnirostris* lineages (Additional file 5). Performing an enrichment analysis to test whether any Gene Ontology (GO) terms [56] were overrepresented among genes with positively selected sites along the passerine branch identified ‘cilium’ (GO:0005929) as the most significantly enriched term (*p* = 8.1×10^-20^; Additional file 6). This term is annotated to three passerine PSGs: coiled-coil domain containing 40 (*CCDC40*), axonemal dynein intermediate chain 2 (*DNAI2*), and cytoplasmic dynein 2 light intermediate chain 1 (*DYNC2LI1*). *DNAI2* protein is a component of respiratory ciliary axonemes and sperm flagella, and human *DNAI2* mutations are associated with respiratory tract dysfunction and infertility [57]. *DYNC2LI1* is present in the mammalian ciliary axoneme [58]. Two further passerine PSGs, namely coiled-coil domain containing 147 (*CCDC147*) and its paralogous gene, coiled-coil domain containing 146 (*CCDC146*), are likely to possess functions related to cilia and spermatazoan flagella (see below), although this is not reflected in current GO annotations.

**Figure 5:**
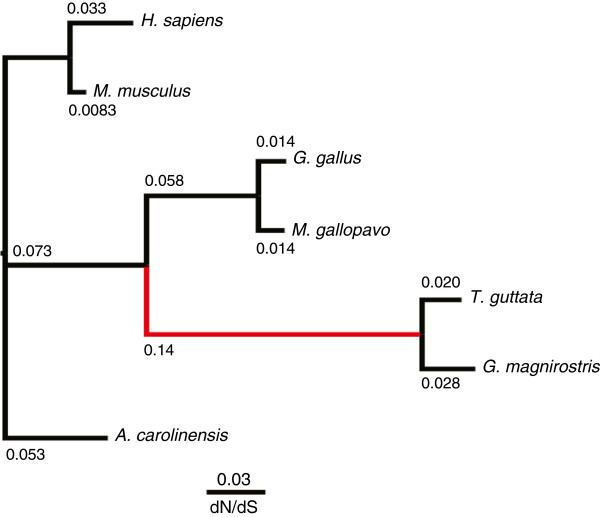
**Gene tree showing the evolution of *CCDC147 *.** Lineage-specific *d*_*N*_*/d*_*S*_ values estimated for the *CCDC147* gene across aminotes. The long passerine branch highlighted in red is inferred to have experienced many events of positive selection.

*CCDC147* is of particular interest as it has evolved unusually rapidly along the passerine branch (Figure 5). It is predicted to harbour 40% more positively selected sites than any other gene inferred for any branch, making it the most pervasively positive selected of all the genes we tested. 27 codon sites in *CCDC147* that are shared by *G. magnirostris* and zebra finch were identified as having been subject to positive selection (posterior probability of >95%), and all 27 of these codon site changes were validated using *G. fortis* sequence data (GenBank entry: AKZB00000000.1 [24]). It is likely that vertebrate *CCDC147* and *CCDC146* homologues encode spermatazoan flagella proteins because its *Chlamydomonas reinhardtii* homologue MBO2 [59,60] is a flagellar protein, and its fruitfly homologues are involved in fertility: *ORY* maps to the *ks-1* fertility factor region, *CG5882* homozygous mutants are sterile [61], and *CG6059* is specifically expressed in the testis. In addition, human *CCDC147* shows the strongest differential expression in the testis ( [62]; http://www.ebi.ac.uk/arrayexpress/experiments/E-GEOD-7307). The positive selection we infer across five passerine genes (*CCDC40*, *DNAI2*, *DYNC2LI1*, *CCDC146* and, most pervasively, *CCDC147*) thus could have been a consequence of sperm competition [63].

## Conclusions

This first genome sequence of a Darwin’s finch has utility beyond the purview of Darwin’s finch biology. Avian species are currently under-sampled as a taxonomic group compared with mammals. Moreover, the passerine order contains over half of all bird species, which equates to approximately 5,000 identified species, almost as many as the total number of mammalian species [64,65]. However, passerines were only represented previously by the genomes of the zebra finch [15] and the flycatcher [66], and our range of genome-scale resources should now facilitate further research into the evolution of this unusual group of passerine birds. Our identification of positively selected genes on the passerine branch not mentioned in previous studies that used only the zebra finch genome sequence [15,44] demonstrates the extra power this additional passerine sequence provides for investigating wider avian biology.

In addition to providing the *G. magnirostris* reads (SRA is SRA061447) and the genome assembly (BioProject accession PRJNA178982), we are now providing gene predictions, orthology relationships, and gene phylogenies generated by this project to browse and to download from http://genserv.anat.ox.ac.uk/clades/vertebrates_geospiza_v3. High quality multiple sequence alignments and regions predicted to have been subject to indel-purifying selection have also been made available from http://wwwfgu.anat.ox.ac.uk/~chrisr/Gmag_data/. Whilst the *G. magnirostris* genome assembly remains incomplete, like many vertebrate genome sequences, it should be finished to high quality once the cost of high quality sequencing is sufficiently reduced. Despite, its draft status the genome assembly provides an important foundation for genetic studies of single genes, and for population genomic studies of most of the genes not just for *G. magnirostris* but also for all other, closely-related, Darwin’s finches. These population approaches should assist in providing an accurate and detailed picture of the demography and phylogenetic history of these finches before and since they arrived on the Galápagos islands approximately 2–3 my ago [10]. Considering the rapid and dramatic morphological and ecological evolution of Darwin’s finches, the comparative study of their genomes will provide valuable insights for speciation genomics, an emerging field of genomics studying genomic-level alterations that accompany processes of divergence and speciation in natural populations [67].

## Methods

### DNA isolation

DNA samples were taken from individual late stage embryos representing three species of Darwin’s finches (*G. magnirostris, G. conirostris* and *G. difficilis*) collected during a field trip to the island of Genovesa (Galápagos) in 2009. The embryonic trunk tissue was preserved in RNAlater solution (Ambion) and treated as fresh tissue with a commercial genomic DNA preparation kit (QIAGEN Genomic DNA Purification Kit). The quality of the obtained gDNA was checked with a NanoDrop Spectrophotometer (ThermoScientific) and Agilent 2100 Bioanalyzer.

### Library construction and sequencing

DNA library construction and sequencing was done at 454-Corporation under the coordination of Timothy Harkins, Jason Affourtit, Clotilde Teiling and Benjamin Boese. DNA libraries were constructed using standard techniques for Roche-454 sequencing. In summary: 3 μg of purified genomic DNA was fractionated into fragments of the targeted size ranges; short adaptors were ligated to each fragments; single stranded fragments were created and immobilized onto specifically designed DNA capture beads; the bead-bound library was emulsified with amplification reagents in water in oil mixture resulting in microreactors containing just (ideally) one bead with one unique sample-library fragment; emulsion beads were submitted to PCR amplification; the emulsion mixture was then broken while the amplified fragments remained bound to their beads; and the DNA-carrying capture beads were loaded onto a PicoTiterPlate device for sequencing. The device was then loaded into the Genome Sequencer system where individual nucleotides are flowed in a fixed order across the open wells and DNA capture beads; complementary nucleotides to the template strand results in a chemiluminescent signal recorded by the CCD camera of the instrument. Roche-454 software was then used to determine the sequence of ~900,000 reads per instrument run – this is done by analyzing a combination of signal intensity and positional information generated across the PicoTiterPlate device.

### Sequencing results

In total twenty-eight long read runs, six runs on 2.5 kbp mate-pair libraries, and six runs on 5 kbp mate-pair libraries were generated. Both Titanium and Titanium XL chemistries were used. Mate-pair libraries in each size range were constructed multiple times, yielding six mate-pair libraries of approximately 5 kbp insert size and an additional five libraries at about 2.5 kbp. Further details on the sequencing data are provided in Additional file 7.

### Genome assembly

Data were obtained from the following 454 runs: 28 small insert “fragment” runs, 6 mate-pair runs covering 5 different ‘3 kbp’ libraries (mean insert size: 2.5 kbp ± 620nt) and 3 mate-pair runs covering 6 ‘8 Kbp’ libraries (mean insert size: 4.9 kbp ± 1.2 kbp). Pyrosequencing reads in SFF format were assembled by the Newbler software version 2.3 using the vendor recommended protocol. Briefly, contigs were generated using the long read data, and mate-pair reads were mapped to the contigs and used to link contigs into scaffolds. In total, 24.4 million reads comprising 7.0 Gbp were used to form contigs and an additional 4.1 million read pairs were used for scaffolding.

The resulting assembly contains 12,958 scaffolds in an estimated genome size of 1254.6Mbp, with a scaffold N50 of 382kbp. The scaffolds comprise 394409 contigs spanning 958.3 Mbp. The coverage distribution has a median at 6.5-fold with a long tail to higher values, which further suggests that some repeat regions may not be fully resolved.

### Whole genome alignments

Chicken and zebra finch genome assemblies (galGal3 and taeGut1 assembly versions) were obtained from UCSC Genome Informatics at http://genome.ucsc.edu (Santa Cruz). The Turkey_2.01 assembly (September 2010) was acquired from Ensembl release 61 at http://www.ensembl.org. LASTZ, available from http://www.bx.psu.edu/miller_lab/, was used to construct the whole genome pairwise alignments, which were subsequently chained and netted using various UCSC utilities [68].

The target genome sequences (chicken, turkey, or zebra finch) when not placed on specific chromosomes were discounted when calculating amounts of aligning sequence; such amounts are thus likely to be conservative estimates. These unplaced sequences were ignored because some sequence in the zebra finch genome assembly is artificially present in two copies, both in assembled chromosomes and in sequence not placed on chromosomes.

Using MULTIZ [69], we combined our zebra finch – *G. magnirostris* whole genome alignments with whole genome alignments between zebra finch – *G.* fortis obtained from UCSC. This resulted in the generation of multiple sequence alignments across zebra finch, *G. magnirostris*, and *G. fortis*.

### Neutral indel model

The Neutral Indel Model (NIM) quantifies the amount of indel-purified sequence (IPS) shared between a species pair. The NIM uses whole genome pairwise alignments to identify inter-gap segments (IGSs) across the genome, and then compares the true distribution of IGSs to the expected neutral geometric distribution that is extrapolated from the distribution of short IGSs inferred from the alignment which are considered to be free from selective constraint. The excess of long IGSs over the neutral expectation are indicative of IPSs containing functional elements. The amount of functional sequence shared between the two species is then estimated by calculating the cumulative lengths of all the IPSs, and then subtracting a correction factor to account for the contribution of neutral sequence to each IPS. Each indel-purified segment is assumed to contain somewhere between *K* and *2 K* bases of neutral sequence depending on the degree of clustering of functional elements, where *K* is the mean number of bases between indels in neutral sequence, which is simply *p*^-1^, where *p* is the indel mutation rate. The upper and lower bound estimates of the amount of IPS are derived using the *K* and *2 K* corrections respectively. The genome is partitioned during the analysis to account for G+C content and sex chromosome biases. Further details of this approach are provided in [27].

The estimated quantities of aligning and indel-purified sequence, and the estimated synonymous divergence between the species are shown in Additional file 8. Frequency histograms of the IGS lengths calculated between the different avian species pairs across all GC content bins are displayed in Additional file 9, Additional file 10, and Additional file 11.

### Gene predictions and orthologue/paralogue assignment

Gene predictions from the *G. magnirostris* genome assembly were made by a computational pipeline, Gpipe, using protein-coding genes from human, chicken, zebra finch as templates [31]. Gene sets for all other seven species were downloaded from Ensembl release 61 (February 2011). Orthologues and paralogues were subsequently assigned using OPTIC [31]. This consists of four steps: (1) orthologues are assigned between pairs of genomes using PhyOP [70] based on a distance metric derived from BLASTP alignments, (2) pairwise orthologues are grouped into clusters, (3) sequences within a cluster are aligned using MUSCLE [71], and (4) phylogenetic tree topologies are estimated using TreeBeST [72] with clusters being split into orthologous groups using the pufferfish *Tetraodon* as the outgroup.

The completeness of these gene sets was examined in two ways. Firstly, the number of simple 1:1 orthologues between human and zebra finch was compared to the number between human and *G. magnirostris*. Secondly, we calculated the number of genes with orthologues predicted in *G. magnirostris* from a set of metazoan single copy genes from Creevey *et al.*[32]. Fifteen of the metazoan single copy genes were excluded from the analysis, since they were retired from the current Ensembl release.

From the OPTIC ortholog sets, a refined ortholog set was constructed of simple 1:1 orthologues shared across human, mouse, chicken, turkey, *G. magnirostris*, zebra finch, and the *Anolis* lizard. False positive predictions of positive selection will be more frequent in poorly aligned or sequence error-prone sequence [73]. Multiple sequence alignments (MSAs) of protein-coding sequence were thus very stringently filtered to remove poorly aligning regions using SEG, GBLOCKS, GUIDANCE [74,75], and further approaches that we describe below. Strict GBLOCKS settings were used (minimum number of sequences for a conserved position=5, minimum number of sequences for a flanking position=6, maximum number of contiguous nonconserved positions=6, minimum length of block=10), only alignment columns with a GUIDANCE score of 1 were kept, and no gaps were allowed. All codons containing a base with a phred quality score of 30 or less, which equates to a 0.1% probability of the base being falsely called, were also excluded. Alignment columns in 15 bp windows were removed when these windows contained greater than 5 substitutions between aligned *G. magnirostris* and zebra finch. Such runs of substitutions may represent sequence or alignment errors. Further alignment columns that lie within 7 codons of previously filtered sequence were also removed since otherwise such codons are enriched in predicted positively predicted sites. Finally, we discarded all genes whose remaining alignment columns numbered fewer than 10% of their predicted numbers of codons, or were less than 100 codons in length. This procedure resulted in a set of “strict” 1:1 orthologues containing 1,452 genes.

### Evolutionary rate analyses

*d*_*S*_, *d*_*N*_, and *d*_*N*_*/d*_*S*_ values were inferred from the filtered MSAs by applying the PAML M2a Maximum-likelihood branch model [76,77]. The branch lengths were then calculated by taking the median values across all genes in the strict orthologue set.

The filtered MSAs and guide trees were also provided as input for the branch-site test for positive selection of Zhang *et al.*[42]. The test identifies genes with particular codons showing evidence of positive selection by comparing a null model, where *d*_*N*_*/d*_*S*_ (ω) is never allowed to exceed 1 (so only negative or neutral evolution is considered), to an alternative model in which some sites on the *G. magnirostris* lineage are allowed to have ω >1 (implying positive selection) (Figure 4a). The test was run twice, and only cases where the two tests converged to within log-likelihood values at or within 0.01 were taken forward for downstream analysis. Subsequently, a likelihood ratio test (LRT) was used to compare the null and alternative model, and a Chi-squared test applied to compare the significance of the LRT scores. The number of positively selected sites in genes inferred to have evolved under positive selection was estimated using a Bayes Empirical Bayes (BEB) approach [43].

It has been suggested that the branch site test of Zhang *et al.*[42] is not statistically robust when the number of substitutions in the MSAs is small [78]. However, this criticism is largely based on the study of Bakewell *et al.*[79] who apply a branch site model across three very closely related primate species. Additionally, it has been suggested that branch-site methods are susceptible to high false positive error when branches assumed to have *d*_*N*_*/d*_*S*_ values less than 1 are in fact evolving rapidly [80]. However, the validity of these criticisms has been challenged [81-83]. The application of the test here across seven diverse amniotes should be robust, since the large number of species, considerable divergence between many species pairs, and the fact that only filtered sequences greater than 100 codons long were tested, mean that there are relatively large numbers of substitutions in each alignment.

### Enrichment analysis

Gene Ontology (GO) annotations for chicken genes were downloaded from http://www.geneontology.org/[56]. GO terms were interpolated to ensure that for each GO term assigned to a gene, all “parental” terms of the GO term were also assigned to that gene. For each GO term, the number of positively selected and non-positively selected sites in genes assigned with that GO term was calculated. A hypergeometric test was then applied in R [84] to calculate a P value for each GO term that represents the probability that the number of positively selected sites observed to be associated with a GO term (or greater number than this) would be seen by chance if positively selected sites were distributed randomly across the genes. A Bonferroni correction was then applied to account for multiple testing [85], producing the adjusted P value that is quoted in the text and in Additional file 6.

### Homology prediction

Homologues of human *CCDC147* were predicted using profile-based iterative searches with the HMMer3 [86], and later the more sensitive HMMer2 [87], algorithms. The algorithms searched for significant sequence similarity between the *CCDC147* sequence and protein sequences in the UniRef50 database [88]. Sequences with significant *E*-value similarity to *CCDC147* where kept, and the *G. magnirostris* and *T. guttata* (and later *G. fortis*) *CCDC147* predicted sequences were added to multiple sequence alignments that were aligned using T-Coffee [89]. Alignments were inspected manually, and lower quality aligning sequences removed, before a phylogenetic tree of the relationship between the sequences was inferred using a Neighbor-joining tree approach [90].

## Competing interests

Researchers from Roche were involved in this project and carried out the DNA library construction and sequencing. Some of the costs of this work were partially covered by Roche.

## Authors’ contributions

CR led the evolutionary rate, positive selection, and neutral indel model analyses, and drafted the manuscript. AD led construction of the genome assembly. MF carried out the G+C content and transposable element analyses. LK contributed gene predictions and orthologue/paralogue assignments. MW analysed substitution patterns in the positively selected genes and contributed helpful discussions on the isochore analysis. CC collected and processed finch material for sequencing. RDE assisted with the application of the test for positive selection. AH assisted with technical aspects of gene predictions and orthologue/paralogue assignment. SM assisted with the neutral indel model analysis. MBH cloned and assisted in the analysis of candidate genes. ME helped initiate the project. CT helped with the genome sequencing. JA participated in experimental design, and technical consultation and coordination of the library construction and sequencing. BB contributed to the genome sequencing. PG and RG wrote the background. JE initiated the project, coordinated the initial sequencing and preliminary analyses, and wrote some of the methods and results sections. AA participated in design and coordination of the study and helped write the background. CPP coordinated the analyses and drafted the manuscript. All authors read and approved the final manuscript.

## Supplementary Material

Additional file 1 The origin of the Darwin's Finch genome project. Click here for file

Additional file 2 **Histograms showing the divergence of transposable element (TE) sequences relative to their consensus sequences for (a) *G. magnirostris* TEs and (b) zebra finch TEs.** Those that are more diverged are more likely to be older. (a) contains TEs defined using a library constructed from the *G. magnirostris* genome assembly, whereas (b) contains TEs defined by RepeatMasker [91]. The paucity of lowly diverged TEs in the *G. magnirostris* genome assembly indicates that it is likely to be most incomplete within repetitive sequence. The figures were generated using scripts from Juan Caballero available at https://github.com/caballero/RepeatLandscape. Click here for file

Additional file 3 **GC content distribution in *G. magnirostris*.** Panel (A) shows the variation of GC content in 3Kb windows along scaffold 10304, the largest scaffold in the assembly. Panel (B) shows the third codon position GC content (GC3) and the equilibrium GC3 (GC3*) content in different vertebrate lineages. The predicted increase in GC content along the Darwin's finch lineage is consistent with the maintenance of GC-rich isochores. Click here for file

Additional file 4 **Base composition properties of *G. magnirostris* positively selected genes.** The genes in bold show a high rate of AT→GC changes. The equilibrium GC content (GC*) was calculated as described by Axelsson *et al.*[92]. Click here for file

Additional file 5 **Positively selected genes along the passerine branch.** P-values of less than 0.01 are highlighted in bold. Click here for file

Additional file 6 Gene Ontology enrichments for positively selected genes along the passerine branch. Click here for file

Additional file 7 Details of 454 Sequencing Runs including length distributions of high quality reads. Click here for file

Additional file 8 Amount of aligning and indel-purified sequence shared between different avian species pairs. Click here for file

Additional file 9 Frequency histograms of inter-gap segments lengths inferred from the *G. gallus* to *G. magnirostris* alignment. Click here for file

Additional file 10 Frequency histograms of inter-gap segments lengths inferred from the *T. guttata* to *G. magnirostris* alignment. Click here for file

Additional file 11 Frequency histograms of inter-gap segments lengths inferred from the *G. gallus* to *T. guttata* alignment. Click here for file
