# Supplementary material for: Insights into the evolution of Darwin’s finches from comparative analysis of the Geospiza magnirostris genome sequence
Source: BMC Genomics. 2013 Feb 12;14:95. doi: 10.1186/1471-2164-14-95 (PMC3575239; doi:10.1186/1471-2164-14-95)
Supplement: Additional file 1 — The origin of the Darwin's Finch genome project. [file 1471-2164-14-95-S1.docx]

**Supplemental File 1**

“Darwin’s Finches” are a model system for the study of various aspects of evolution and development. In 2008 we commenced a project to sequence the genomes of some of these species – inspired by the (then) upcoming celebration of the 200^th^ anniversary of the birth of Charles Darwin (which was in February 2009). The project started with a brief discussion at the AGBT meeting in 2008 and then via an email conversation between Jonathan Eisen and Jason Affourtit about the possibility of a collaboration involving the 454 company (which was looking for projects to highlight the power of its then relatively new 454 sequencing machines). After further discussions between Jonathan Eisen, his brother Michael Eisen (who separately had become interested in Darwin’s finches) and people from 454, it was decided that this was a potentially good project for a scientific and marketing collaboration.

In these conversations it was determined that the most likely limiting factor would be access to DNA from the finches. This was an issue due to the Galapagos Islands being a National Park in Ecuador and also a World Heritage site within which collection of samples for any type of research is highly regulated. Thus, Jonathan Eisen made contact with Peter and Rosemary Grant – prominent researchers working on the finches – and who Eisen had discussed sequencing the finch genomes in the early 2000s. In that previous conversation it was determined that the sequencing would be too expensive to carry out without a major fundraising effort. However, with the advent of “next generation” sequencing methods, such as 454, the total costs of such a project would be much lower. In conversations with the Grants they offered to explore whether anyone had sufficient amounts of DNA (or access to samples), which would be needed for genome library construction. Subsequently they identified Arhat Abzhanov from Harvard as someone most likely to have samples, as well as permission to perform DNA-based work on them, from many of the finch species.

Abzhanov offered to provide samples from three key species (large ground finch *Geospiza magnirostris*, large cactus finch *G. conirostris* and sharp-billed finch *G. difficilis*) and DNA was sent to Roche-454 for sequencing in July of 2008. In August, the first “test” sequence data was provided from *Geospiza magnirostris*. A plan was then made to generate additional data and Roche offered to do the sequencing at their center at a discount. Funds were raised by Jonathan Eisen, Greg Wray, Monica Riley, and others to pay for the sequencing and over the next year or so, three sequencing bursts were conducted at Roche-454. As the most deeply sequenced species was *G. magnirostris*, an assembly was generated for this species.
