# Supplementary figures and images for: Insights into the evolution of Darwin’s finches from comparative analysis of the Geospiza magnirostris genome sequence
Source: BMC Genomics. 2013 Feb 12;14:95. doi: 10.1186/1471-2164-14-95 (PMC3575239; doi:10.1186/1471-2164-14-95)

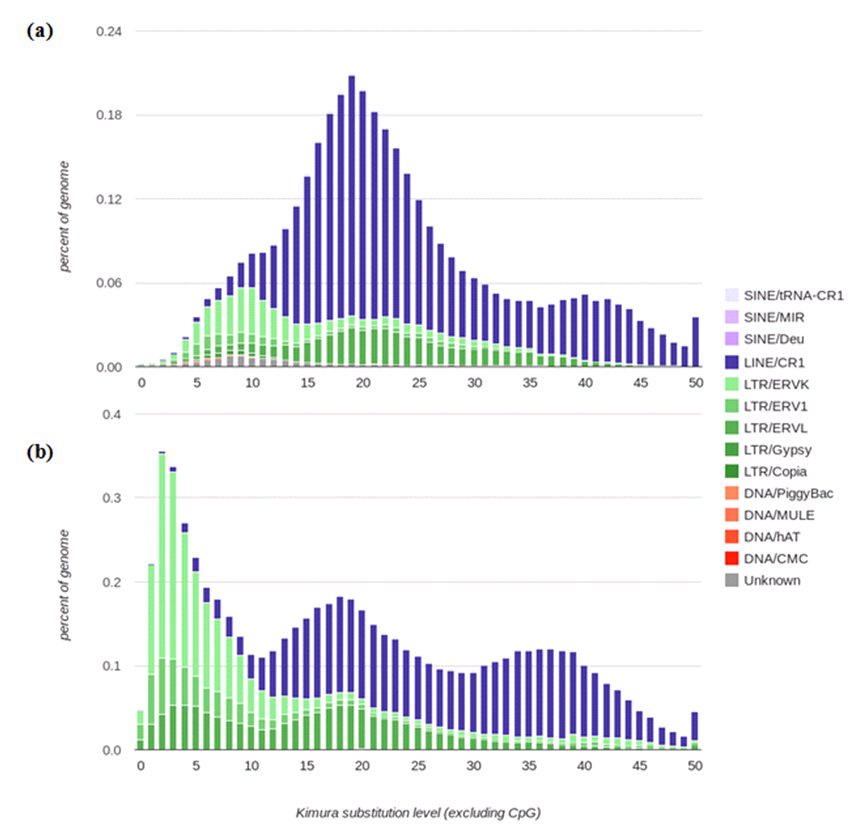

Supplement: Additional file 2 — Histograms showing the divergence of transposable element (TE) sequences relative to their consensus sequences for (a) G. magnirostris TEs and (b) zebra finch TEs. Those that are more diverged are more likely to be older. (a) contains TEs defined using a library constructed from the G. magnirostris genome assembly, whereas (b) contains TEs defined by RepeatMasker [91]. The paucity of lowly diverged TEs in the G. magnirostris genome assembly indicates that it is likely to be most incomplete within repetitive sequence. The figures were generated using scripts from Juan Caballero available at https://github.com/caballero/RepeatLandscape. [file 1471-2164-14-95-S2.png]
