# Supplementary material for: Insights into the evolution of Darwin’s finches from comparative analysis of the Geospiza magnirostris genome sequence
Source: BMC Genomics. 2013 Feb 12;14:95. doi: 10.1186/1471-2164-14-95 (PMC3575239; doi:10.1186/1471-2164-14-95)
Supplement: Additional file 4 — Base composition properties of G. magnirostris positively selected genes. The genes in bold show a high rate of AT→GC changes. The equilibrium GC content (GC*) was calculated as described by Axelsson et al.[92]. [file 1471-2164-14-95-S4.docx]

| **Short gene name** | **Ensembl gene ID of chicken 1:1 ortholog** | **Length of filtered aligning sequence (bp)** | **GC content proportion** | **Number of GC**→**AT Mutations** | **Number of AT**→**GC Mutations** | **Darwin’s finch equilibrium GC content (GC*)** |
| --- | --- | --- | --- | --- | --- | --- |
| *FKBP6* | ENSGALG00000000837 | 340 | 0.539 | 3 | 4 | 0.609 |
| *MFF* | ENSGALG00000003079 | 513 | 0.517 | 2 | 2 | 0.517 |
| *ASB6* | ENSGALG00000004378 | 362 | 0.624 | 2 | 1 | 0.453 |
| *SART3* | ENSGALG00000004887 | 924 | 0.524 | 2 | 1 | 0.270 |
| ***UBP47*** | **ENSGALG00000005569** | **1262** | **0.448** | **2** | **20** | **0.890** |
| *TRAF7* | ENSGALG00000005767 | 662 | 0.568 | 5 | 4 | 0.513 |
| *XDH* | ENSGALG00000008701 | 2353 | 0.491 | 14 | 19 | 0.567 |
| ***E1BY77*** | **ENSGALG00000008909** | **785** | **0.497** | **2** | **9** | **0.816** |
| *F1N8A7* | ENSGALG00000010043 | 622 | 0.469 | 0 | 3 | N/A |
| *P2RY1* | ENSGALG00000010357 | 763 | 0.482 | 9 | 1 | 0.0939 |
| *F1NDU4* | ENSGALG00000011096 | 424 | 0.453 | 2 | 1 | 0.293 |
| *PRKAG3* | ENSGALG00000011360 | 657 | 0.556 | 3 | 3 | 0.556 |
| *ANO10* | ENSGALG00000011513 | 694 | 0.433 | 7 | 5 | 0.353 |
| ***IGF2R*** | **ENSGALG00000011621** | **2501** | **0.446** | **8** | **27** | **0.731** |
| *F1NIP9* | ENSGALG00000012138 | 357 | 0.336 | 0 | 1 | N/A |
| *LRR1* | ENSGALG00000012230 | 278 | 0.607 | 0 | 4 | N/A |
| *C7orf25* | ENSGALG00000012333 | 737 | 0.482 | 5 | 12 | 0.691 |
| *Q9DEH4* | ENSGALG00000012495 | 5369 | 0.509 | 26 | 37 | 0.596 |
| *ARSK* | ENSGALG00000014672 | 488 | 0.391 | 4 | 5 | 0.445 |
| *F1NR67*  *(POU1F1)* | ENSGALG00000015495 | 411 | 0.533 | 2 | 5 | 0.740 |
| *E1BV11* | ENSGALG00000016811 | 389 | 0.388 | 3 | 2 | 0.297 |
