# Supplementary material for: Insights into the evolution of Darwin’s finches from comparative analysis of the Geospiza magnirostris genome sequence
Source: BMC Genomics. 2013 Feb 12;14:95. doi: 10.1186/1471-2164-14-95 (PMC3575239; doi:10.1186/1471-2164-14-95)
Supplement: Additional file 5 — Positively selected genes along the passerine branch. P-values of less than 0.01 are highlighted in bold. [file 1471-2164-14-95-S5.docx]

| **Short gene name** | **Ensembl gene ID of chicken 1:1 ortholog** | **P-value that gene is under positive selection** | **Number of codon sites inferred to be under positive selection with p<0.1** |
| --- | --- | --- | --- |
| *RNF207* | ENSGALG00000000710 | 0.015 | 1 |
| *FOXRED1* | ENSGALG00000001033 | 0.013 | 1 |
| *E1C8X7* | ENSGALG00000001226 | 0.040 | 1 |
| *SAMHD1* | ENSGALG00000001231 | **0.0055** | 6 |
| *F1NVV4* | ENSGALG00000002466 | 0.039 | 0 |
| *F1P582* | ENSGALG00000002490 | 0.038 | 2 |
| *GMPPB* | ENSGALG00000002500 | 0.016 | 0 |
| *E1BQB6* | ENSGALG00000002891 | 0.011 | 1 |
| *PMS2* | ENSGALG00000003430 | 0.030 | 1 |
| *SGMS1* | ENSGALG00000003701 | **0.0077** | 1 |
| *TTYH3* | ENSGALG00000004310 | 0.043 | 1 |
| *DNAI2* | ENSGALG00000004495 | **0.0072** | 19 |
| *E1BQH2* | ENSGALG00000004813 | 0.043 | 1 |
| *F1N867* | ENSGALG00000005693 | 0.047 | 1 |
| *SLC52A3* | ENSGALG00000006194 | 0.018 | 0 |
| *LRRC33* | ENSGALG00000006402 | 0.037 | 11 |
| *C12H3orf19* | ENSGALG00000006459 | 0.045 | 1 |
| *SETD5* | ENSGALG00000006571 | 0.033 | 1 |
| *CCDC40* | ENSGALG00000007042 | **0.0000017** | 10 |
| *FAM46D* | ENSGALG00000007157 | 0.018 | 1 |
| *CLEC16A* | ENSGALG00000007167 | 0.030 | 2 |
| *PDIA1* | ENSGALG00000007233 | **0.0053** | 1 |
| *Q5ZJK3* | ENSGALG00000007651 | 0.019 | 2 |
| *ARMC9* | ENSGALG00000007691 | **0.0082** | 25 |
| *PSME4* | ENSGALG00000008163 | 0.046 | 1 |
| *TRIM36* | ENSGALG00000008188 | 0.035 | 3 |
| *CCDC146* | ENSGALG00000008309 | 0.013 | 15 |
| *CCDC147* | ENSGALG00000008417 | **0.00096** | 36 |
| *ATL2* | ENSGALG00000008515 | **0.000013** | 2 |
| *XDH* | ENSGALG00000008701 | 0.011 | 0 |
| *E1BRU9* | ENSGALG00000008813 | 0.014 | 1 |
| *LBR* | ENSGALG00000009305 | **0.0098** | 1 |
| *LRRC34* | ENSGALG00000009402 | **0.00010** | 7 |
| *RAD51B* | ENSGALG00000009491 | 0.038 | 0 |
| *DYNC2LI1* | ENSGALG00000009954 | **0.0019** | 1 |
| *AKR1A1* | ENSGALG00000010244 | 0.043 | 1 |
| *SLC4A4* | ENSGALG00000011604 | 0.029 | 1 |
| *Q7ZSX8* | ENSGALG00000011715 | 0.018 | 1 |
| *DTX3L* | ENSGALG00000012075 | 0.040 | 1 |
| *CHST11* | ENSGALG00000012698 | 0.018 | 1 |
| *FUCA2* | ENSGALG00000013773 | **0.000023** | 2 |
| *Q5ZL94* | ENSGALG00000015916 | 0.022 | 1 |
| *DCA13* | ENSGALG00000016073 | 0.046 | 1 |
| *Q5ZLT2* | ENSGALG00000016323 | **0.0033** | 1 |
| *F1NHR2* | ENSGALG00000016558 | **0.0026** | 1 |
| *ATM* | ENSGALG00000017159 | **0.0046** | 2 |
| *GPR162* | ENSGALG00000022926 | 0.030 | 1 |
