# Supplementary material for: Insights into the evolution of Darwin’s finches from comparative analysis of the Geospiza magnirostris genome sequence
Source: BMC Genomics. 2013 Feb 12;14:95. doi: 10.1186/1471-2164-14-95 (PMC3575239; doi:10.1186/1471-2164-14-95)
Supplement: Additional file 6 — Gene Ontology enrichments for positively selected genes along the passerine branch. [file 1471-2164-14-95-S6.docx]

| **GO term** | **GO term description** | **Enrichment P-Value** |
| --- | --- | --- |
| GO:0005929 | cilium | 8.14e-20 |
| GO:0071844 | cellular component assembly  at cellular level | 3.63e-09 |
| GO:0022607 | cellular component assembly | 8.07e-08 |
| GO:0048858 | cell projection morphogenesis | 1.55e-07 |
| GO:0044085 | cellular component biogenesis | 2.48e-07 |
| GO:0044463 | cell projection part | 3.17e-07 |
| GO:0032990 | cell part morphogenesis | 6.66e-07 |
| GO:0042995 | cell projection | 1.25e-06 |
| GO:0030030 | cell projection organization | 2.25e-05 |
| GO:0000902 | cell morphogenesis | 0.000919 |
| GO:0048646 | anatomical structure formation involved in morphogenesis | 0.00135 |
| GO:0007017 | microtubule-based process | 0.00923 |
| GO:0043623 | cellular protein complex assembly | 0.0177 |
