# Supplementary material for: Insights into the evolution of Darwin’s finches from comparative analysis of the Geospiza magnirostris genome sequence
Source: BMC Genomics. 2013 Feb 12;14:95. doi: 10.1186/1471-2164-14-95 (PMC3575239; doi:10.1186/1471-2164-14-95)

**a) Sequencing Results Run 1**

| **Length Range** | **Number of Sequences** |
| --- | --- |
| 0:100 | 96016 |
| 100:200 | 115557 |
| 200:300 | 146226 |
| 300:400 | 220452 |
| 400:500 | 268893 |
| 500:600 | 86320 |
| 600:700 | 57 |
| 700:800 | 1 |

        Total number of bases:  305397463

        Total GC count:                 128579775

        GC %:                           42.10 %

        Total number of sequences:      933522

**b) Sequencing Results Set 2 Statistics**

| **Run** | **Region** | **SID** | **Name** | **HQ Reads** | **Bases** | **Avg Read Length** | **Keypass** |
| --- | --- | --- | --- | --- | --- | --- | --- |
| RunID26097618 | 1 | 9578 | *G.magnirostris* | 533,836 | 170,364,996 | 319 | 74.2% |
| RunID26097618 | 2 | 9578 | *G.magnirostris* | 459,687 | 140,086,587 | 305 | 63.1% |
| RunID26098233 | 1 | 9580 | *G.difficilis* | 586,585 | 221,537,440 | 378 | 74.9% |
| RunID26098233 | 2 | 9580 | *G.difficilis* | 516,152 | 199,682,960 | 387 | 79.7% |
| RunID26128441 | 1 | 9578 | *G.magnirostris* | 637,913 | 235,013,322 | 368 | 86.2% |
| RunID26128441 | 2 | 9578 | *G.magnirostris* | 586,998 | 223,288,245 | 380 | 85.3% |
| RunID26128452 | 1 | 9578 | *G.magnirostris* | 600,366 | 249,213,979 | 415 | 82.2% |
| RunID26128452 | 2 | 9578 | *G.magnirostris* | 611,533 | 247,086,590 | 404 | 80.1% |
| RunID26128457 | 1 | 9578 | *G.magnirostris* | 597,511 | 208,348,475 | 349 | 79.0% |
| RunID26128457 | 2 | 9578 | *G.magnirostris* | 617,661 | 223,994,938 | 363 | 81.2% |

**c) Sequencing Results Set 2 read length distributions for different runs**


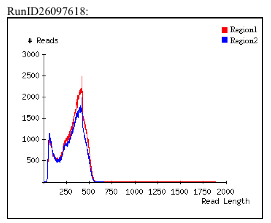


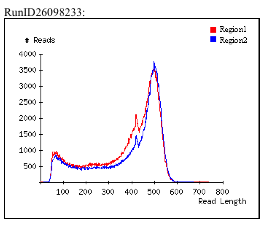


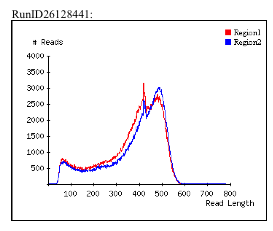


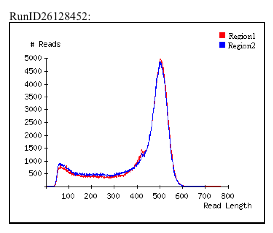


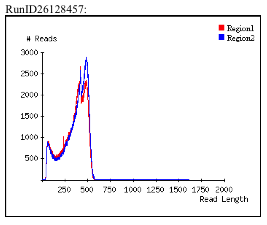


**d) Sequencing Results Set 3 Statistics**

| **Run** | **Region** | **SID** | **Name** | **HQ Reads** | **Bases** | **Avg Read Length** |
| --- | --- | --- | --- | --- | --- | --- |
| RunID26703047 | 1 | 10653 | *G.magnirostris* | 487,667 | 183,491,936 | 376 |
| RunID26703047 | 2 | 10653 | *G.magnirostris* | 511,010 | 191,736,388 | 375 |
| RunID26699701 | 1 | 10653 | *G.magnirostris* | 566,875 | 180,431,221 | 318 |
| RunID26699701 | 2 | 10653 | *G.magnirostris* | 566,203 | 178,897,605 | 316 |
| RunID26699628 | 1 | 10653 | *G.magnirostris* | 488,439 | 192,145,285 | 393 |
| RunID26699628 | 2 | 10653 | *G.magnirostris* | 449,884 | 165,564,988 | 368 |
| RunID26699564 | 1 | 10653 | *G.magnirostris* | 365,274 | 145,252,121 | 398 |
| RunID26699564 | 2 | 10653 | *G.magnirostris* | 360,435 | 143,698,100 | 399 |
| RunID26699561 | 1 | 10653 | *G.magnirostris* | 366,112 | 149,207,137 | 408 |
| RunID26699561 | 2 | 10653 | *G.magnirostris* | 362,251 | 148,082,461 | 409 |
| RunID26731018 | 1 | 10653 | *G.magnirostris* | 489,906 | 181,879,314 | 371 |
| RunID26731018 | 2 | 10653 | *G.magnirostris* | 497,915 | 191,199,753 | 384 |
| RunID26651149 | 1 | 10653 | *G.magnirostris* | 485,161 | 150,929,173 | 311 |
| RunID26651149 | 2 | 10653 | *G.magnirostris* | 517,839 | 173,161,226 | 334 |
| RunID26641211 | 1 | 10653 | *G.magnirostris* | 554,726 | 177,197,271 | 319 |
| RunID26641211 | 2 | 10653 | *G.magnirostris* | 541,253 | 176,740,305 | 327 |
| RunID26625034 | 1 | 10653 | *G.magnirostris* | 523,937 | 204,855,319 | 391 |
| RunID26625034 | 2 | 10653 | *G.magnirostris* | 527,034 | 205,620,475 | 390 |
| SID10653 | 1 | 10653 | *G.magnirostris* | 573,265 | 186,215,974 | 325 |
| SID10653 | 2 | 10653 | *G.magnirostris* | 579,094 | 189,950,986 | 328 |
| Summary of all runs | N/A | 10653 | *G.magnirostris* | 9,814,280 | 3,516,257,038 | N/A |

**e) Sequencing Results Set 3 read length distributions for different runs**

RunID26703047  

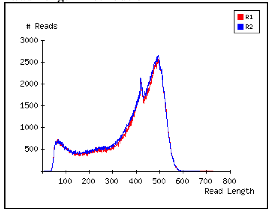


RunID26699701


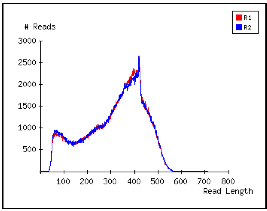


RunID26699628


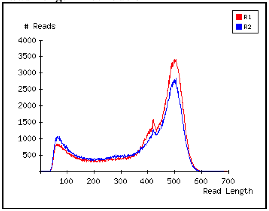


RunID26699564


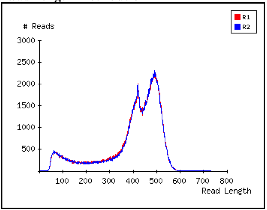


RunID26699561


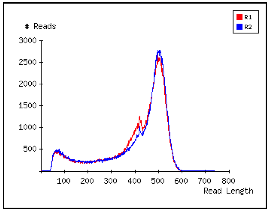


RunID26731018


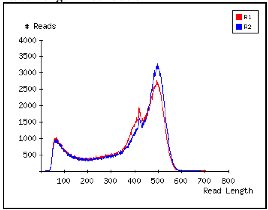


RunID26651149


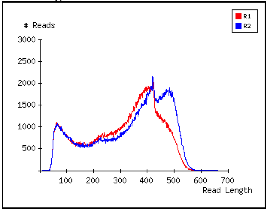


RunID26641211


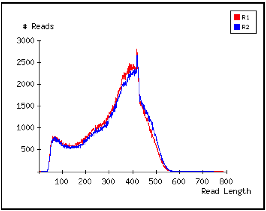


RunID26625034


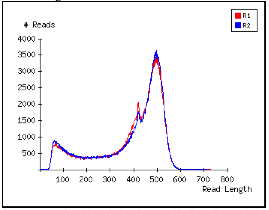


SID10653


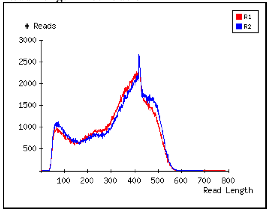

Supplement: Additional file 7 — Details of 454 Sequencing Runs including length distributions of high quality reads. [file 1471-2164-14-95-S7.docx]
