# Supplementary material for: Insights into the evolution of Darwin’s finches from comparative analysis of the Geospiza magnirostris genome sequence
Source: BMC Genomics. 2013 Feb 12;14:95. doi: 10.1186/1471-2164-14-95 (PMC3575239; doi:10.1186/1471-2164-14-95)
Supplement: Additional file 8 — Amount of aligning and indel-purified sequence shared between different avian species pairs. [file 1471-2164-14-95-S8.docx]

| **Species**  **Pair** | **Aligning**  **Sequence (Mb)** | **Indel-purified sequence (Mb)** | | **Divergence**  **(dS)** |
| --- | --- | --- | --- | --- |
|  |  | **Lower** | **Upper** |  |
| *G. gallus* – *G. magnirostris* | 569 | 80 | 102 | 0.24 |
| *G. gallus* – *T. guttata* | 648 | 96 | 120 | 0.23 |
| *T. guttata* – *G. magnirostris* | 823 | 120 | 179 | 0.093 |
